# Piezoelectricity and Thermophysical Properties of Ba_0.90_Ca_0.10_Ti_0.96_Zr_0.04_O_3_ Ceramics Modified with Amphoteric Nd^3+^ and Y^3+^ Dopants

**DOI:** 10.3390/ma16062369

**Published:** 2023-03-15

**Authors:** Yongshang Tian, Mingyang Ma, Shuiyun Li, Junli Dong, Xiang Ji, Haitao Wu, Jinshuang Wang, Qiangshan Jing

**Affiliations:** 1 College of Chemistry and Chemical Engineering, Henan Key Laboratory of Utilization of Non-Metallic Mineral in the South of Henan, Xinyang Normal University, Xinyang 464000, China; 2 School of Materials and Chemical Technology, Tokyo Institute of Technology, Tokyo 152-8552, Japan; 3 School of Environmental and Material Engineering, Yantai University, Yantai 264005, China

**Keywords:** BCTZ-NY*x* ceramics, oxygen vacancies, activation energy, piezoelectric properties, thermophysical performance

## Abstract

Lead-free barium calcium titanate zirconate (BCTZ) ceramics doped with a single rare-earth element generally exhibit excellent piezoelectric properties. However, their electrical properties deteriorate at an excessive dopant content, limiting their application. In this study, amphoteric neodymium (Nd^3+^) and yttrium (Y^3+^)-codoped BCTZ-NY*x* ceramics were synthesized via a solid-state reaction at 1240 °C. The influences of the Y^3+^ content (*x*) on the structural features, electrical properties, mechanical properties, and thermophysical properties were investigated. At a small *x* (<0.18 mol%), Y^3+^ could enhance the fracture strength and electrical properties by eliminating oxygen vacancies, defect dipoles, and/or structural defects. However, the outstanding performance deteriorated with excessive *x*. Additionally, the mechanism of the defect chemistry at different *x* was deduced. At an yttrium content of 0.18 mol%, the ceramic exhibited high piezoelectricity and ferroelectricity with low domain-switching activation energy (*E*_a_ = 0.401 eV), indicating that it could replace commercial lead-based piezoelectric ceramics.

## 1. Introduction

Piezoelectric response materials are widely used in commercial ultrasonic motors, actuators, and energy-harvesting devices because of their electrical-to-mechanical energy interconversion [1,2,3]. Among the piezoelectric materials, lead-based perovskite ferroelectrics exhibit state-of-the-art piezoelectric properties and temperature stability [4]. However, as lead-containing materials are highly toxic and have an adverse environmental impact, developing safe lead-free alternatives in commercial products was urgent according to the restricted regulations and laws formulated in recent years [5,6]. The key to developing viable lead-free materials is to ensure that they possess a similar morphotropic phase boundary (MPB) in their structure or analogical piezoelectric properties to those of lead-based materials. Some selected lead-free materials with excellent piezoelectric properties and a polymorphic phase transition (PPT) have been identified, and calcium and zirconate-codoped BaTiO_3_ (BCTZ) materials with an MPB in the structure, similar to that of lead-based materials, have attracted widespread attention [7,8,9,10].

In addition to having an MPB, BCZT-based ceramics feature outstanding piezoelectric properties stemming from their good piezoelectric response owing to many dipole polarisation rotation directions with a low activation energy [11]. Thus, a large number of studies have focused on domain configuration, defect dipole regulation, and phase boundary construction for achieving excellent piezoelectric properties [12,13]. Rare-earth elements exhibit an amphoteric character in the perovskite structure (ABO_3_) because of their ionic valence state and ionic radius, and they are generally used to dope BCZT-based materials to elevate their electrical properties [14,15]. According to the defect chemistry, the doping of a small amount of a rare-earth element that acts as a donor in the ABO_3_ structure could decrease the volume of oxygen vacancies and structural defects. However, the electrical properties recede with excessive single rare-earth elements, which act as acceptors [16]. Consequently, BCZT-based materials codoped with two or more rare-earth elements are being explored as potential materials with excellent piezoelectric properties. For example, Zuo et al. reported that BCZT ceramics codoped with Er and Yb exhibit excellent upconversion emission and piezoelectric properties [17]. Hamza reported that BCZT ceramics codoped with Nd, Y, and Gd have excellent ferroelectric and dielectric properties [18]. Tian et al. reported that Yb,Dy-codoped BCZT ceramics exhibit a high piezoelectric response and ferroelectricity [19]. Li et al. reported a high piezoelectric constant and electromechanical coupling coefficient for Ce,Y-codoped BCZT ceramics [20]. Batoo et al. reported that Gd,Nb-codoped BCZT ceramics exhibit good photoluminescence and dielectric properties [21].

BCZT ceramics singly doped with a small quantity of Nd or Y are known to exhibit outstanding piezoelectricity, ferroelectricity, and reliable temperature stability [22,23,24], but Nd and Y-codoped BCZT ceramics have not been reported so far, although they are expected to exhibit significantly enhanced electrical performance, i.e., the experimental results in this work show that the piezoelectric constant (284 pC/N) and remnant polarisation (~18 μC/cm^2^) of the codoped ceramics were more excellent than the results (206 pC/N and ~9 μC/cm^2^) of single Nd or Y doped BCZT ceramics. Moreover, the mechanism by which the electrical properties are influenced by the defect dipoles and oxygen vacancies has rarely been confirmed experimentally. Additionally, the thermophysical properties and fracture strength of a ceramic can play a vital role in commercial electronic components because a good degree of matching between the substrate material and BCZT-based matrix ceramic is essential [25]; however, studies on these aspects have also been rarely reported.

In this study, amphoteric Nd^3+^,Y^3+^-codoped Ba_0.90_Ca_0.10_Ti_0.96_Zr_0.04_O_3_ ceramics were prepared and researched. The influences of the Y^3+^ dopant contents on the ceramic phase structure features, ferroelectric properties, dielectric properties, piezoelectric response, fracture strength, and thermal expansion coefficient were investigated. The mechanism of the defect chemistry at different Y^3+^ contents was deduced through a series of measurements. Moreover, a high piezoelectric response was confirmed when the defect dipoles and oxygen vacancies were reduced in the structure because of a low domain-switching activation energy.

## 2. Experimental Procedure

### 2.1. Preparation

Ceramic samples with a composition of Ba_0.90_Ca_0.10_Ti_0.96_Zr_0.04_O_3_-0.05 mol%Nd-*x*Y (abbreviated as BCTZ-NY*x*, *x* = 0, 0.06, 0.12, 0.18, 0.24, and 0.30 mol%) were synthesised via a conventional solid-state reaction with BCTZ-NY*x* nanoparticles that were prepared by a modified Pechini polymeric precursor method. Y(NO_3_)_3_·6H_2_O, Ba(CH_3_COO)_2_, Ti(OC_4_H_9_)_4_, Ca(NO_3_)_2_·4H_2_O, Zr(NO_3_)_4_·5H_2_O, Nd(NO_3_)_3_·6H_2_O, citric acid, and ethylene glycol were used as the main raw materials to synthesise the as-prepared nanoparticles, and the details of the synthesis may be found in our previous reports [16,26]. With a little 2.0 wt.% polyvinyl alcohol water solution, the as-prepared nanoparticles were mixed uniformly and pressed at 150 MPa pressure into green pellets uniaxially. Then, the binder was removed through combustion at 1240 °C for 4 h in the muffle furnace to produce a batch of green pellets. The green pellets were subsequently polished and then coated with silver paste to serve as electrodes (5 mm in diameter) for evaluating their electrical properties. Thereafter, the electrodes were removed from parallel disc-shaped sample surfaces using an automatic polish-grinding machine, and the ceramic samples were cut into approximately 10 mm × 4 mm × 0.8 mm specimens for evaluating their thermophysical performance and fracture strength.

### 2.2. Characterisation

X-ray diffraction (XRD) was conducted using Cu-*Kα* radiation (Rigaku Smartlab 9kW, Japan) under a 2*θ* scanned rate of 0.05°/s to examine the structures of the BCTZ-NY*x* ceramics. Raman spectrum instrument (Horiba Jobin Yvon, France) was performed to identify the message of crystal symmetry and structural defects of the ceramics. The cross-sectional microstructures of the ceramics were studied by scanning electron microscopy (SEM; S4800, Japan). The densities of the ceramics were calculated according to the Archimedes immersion principle; a precision electronic balance (ED–124S, China) was conducted to analyse the samples weight. X-ray photoelectron spectroscopy (XPS; K-ALPHA, UK) was performed to characterise the ceramic elements electronic and valence states. The dielectric behaviour (relative permittivities and loss tangents) was assessed with various frequencies and temperatures (25 to 200 °C) through a dielectric testing instrument (HDMS-1000V, China) coupled with a LCR instrument (WK-6500B, UK). The hysteresis loops of the ceramics polarisation-electric field (*P*-*E*) were obtained by a radiant precision workstation (RTI-Multiferroic II, USA), and the loops of the ceramics strain-electric field (*S*-*E*) were gained by an optical reflectance sensor (MTI-2100, USA), which could be used to calculate the piezoelectric coefficients (*d*_33_*). An impedance analyser (E4990A, USA) was performed to characterise the planar vibration electromechanical coupling (*k*_p_) and mechanical quality (*Q*_m_) factors. The piezoelectric constants (*d*_33_) were observed using a quasistatic piezoelectricity testing instrument (HCYD-800, China). A bending tester (XD-300N, China) was used to measure the ceramic fracture strengths. The thermal expansion performance was measured by a dilatometer (DIL 402, Germany).

## 3. Results and Discussion

### 3.1. Phases and Structures

**Figure 1:**
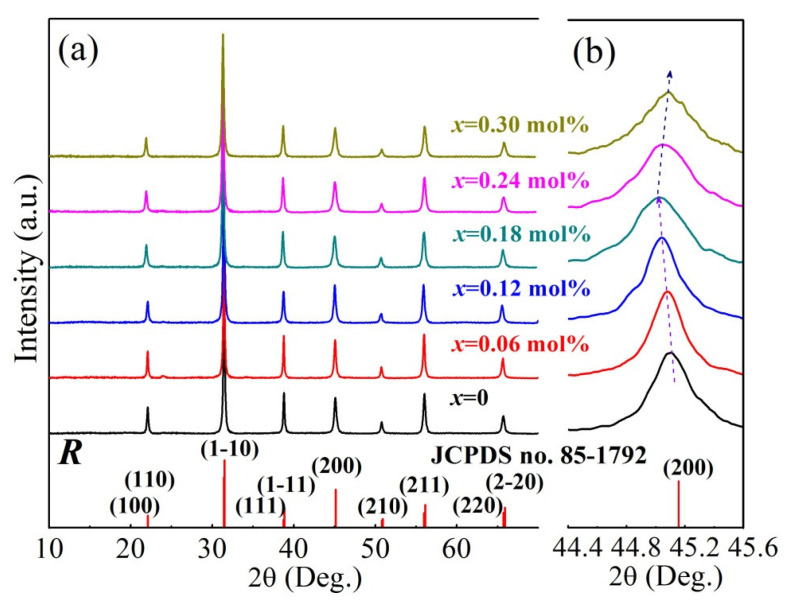
(**a**) XRD patterns for the BCTZ-NY*x* ceramics synthesized with various yttrium contents (*x*) and (**b**) selected enlarged regions of the XRD patterns.

Figure 1 shows the room-temperature XRD patterns of the BCTZ-NY*x* ceramics prepared with variable yttrium dopant contents (*x*) in the 2*θ* range of 10 to 70°. The XRD patterns exhibit no apparent peaks of secondary phases and contain the peaks of an almost pure perovskite phase with a rhombohedral (*R*) structure (JCPDS No. 85-1792) and puny CaTiO_3_ phase peaks around 23° that are indexed with JCPDS No. 75-2099. The result of the Rietveld-refined XRD pattern for the BCTZ-NY*x* ceramic with 0.18 mol% yttrium (Figure S1) confirmed this inference. The results suggest that the doping of yttrium did not severely disturb the phase evolution and that the crystal lattice was completely impregnated with yttrium ions, which was further confirmed by Raman spectroscopy (Figure S2) [27]. To clearly demonstrate the change in the diffraction peak position of the BCTZ-NY*x* ceramic with the increment of yttrium content, the fine XRD patterns in the 2*θ* range of 44.4 to 45.6° are enlarged in Figure 1b. It could be detected that the (200) diffraction peaks first turn to the lower 2*θ* region and then to the higher 2*θ* region with an increment *x* further; this phenomenon could be associated with variations in the crystalline interplanar spacing of the ceramic [28]. At a low content, Y^3+^ (ionic radius, 0.106 nm) replaced the A site Ca^2+^ (0.134 nm) or Ba^2+^ (0.161 nm) species in the ABO_3_ structure, leading to an enlarged crystalline interplanar spacing, as determined from the Bragg equation. The interplanar spacing decreased upon excessive yttrium doping because Y^3+^ started to substitute the B site Zr^4+^ (0.074 nm) or Ti^4+^ (0.068 nm) species in the structure. Table 1 shows the detailed lattice parameters (*a*, *b*, *c*, and *Axial angle*) refined by a Rietveld fitting procedure such as Figure S1, and they are consistent with the above conclusions. Additionally, the intensity of (200) diffraction peaks first enhanced and then reduced with the increment of yttrium dopant contents, suggesting that the crystallinity of the ceramic increased at first and then decreased.

**Figure 2:**
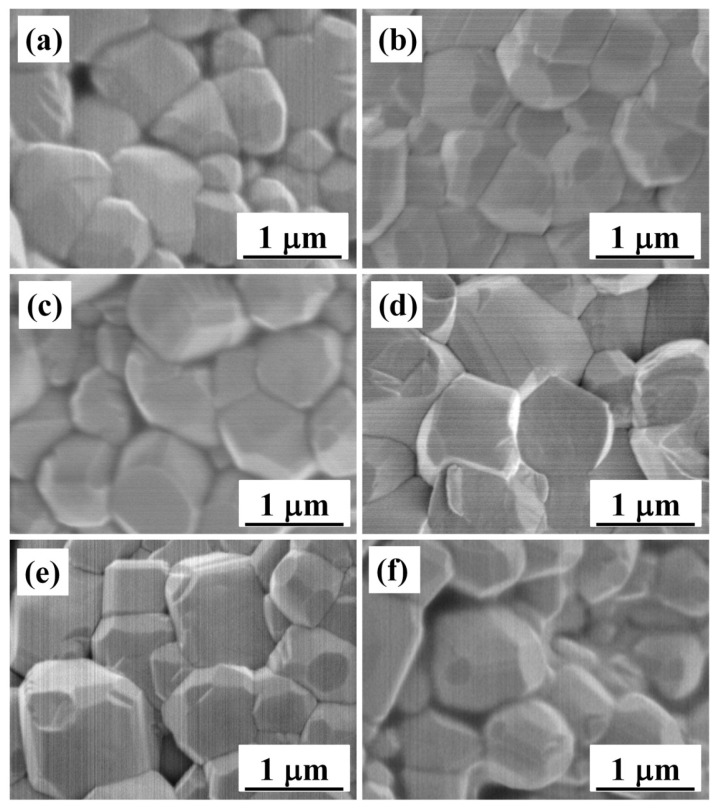
SEM images of the BCTZ-NY*x* ceramics fracture morphology with various yttrium contents (*x*) of (**a**) 0, (**b**) 0.06, (**c**) 0.12, (**d**) 0.18, (**e**) 0.24, and (**f**) 0.30 mol%.

To demonstrate the effects of the yttrium substitution (*x*) on the densification and grain size of the BCTZ-NY*x* ceramic, the calculated density (*ρ*) and relative density (*ρ*_r_) values of the samples with different *x* are presented in Table 1, and the SEM images message of the fracture morphologies are displayed in Figure 2. All samples presented adequate densification (*ρ*_r_, ~96%), suggesting that the preparation process used in this study is suitable and effective. The regular grains grew adequately and were packed closely, forming a highly densified microstructure. The average grain size was ~1 μm for all samples as calculated by the linear intercept method, which implies that the grain size was fine and stable and that the yttrium substitution did not have an apparent effect on the grain size. Cavities were formed in the ceramic structure with excessive yttrium doping, because of which the densification of the ceramic initially increased slightly and subsequently decreased with increasing *x*. Moreover, a more impacted structure with transgranular fracture of the BCTZ-NY*x* ceramic with 0.18 mol% yttrium (Figure 2d) suggested a high fracture strength (K) (96.3 MPa, Table S1) [29].

### 3.2. Dielectric Properties

**Figure 3:**
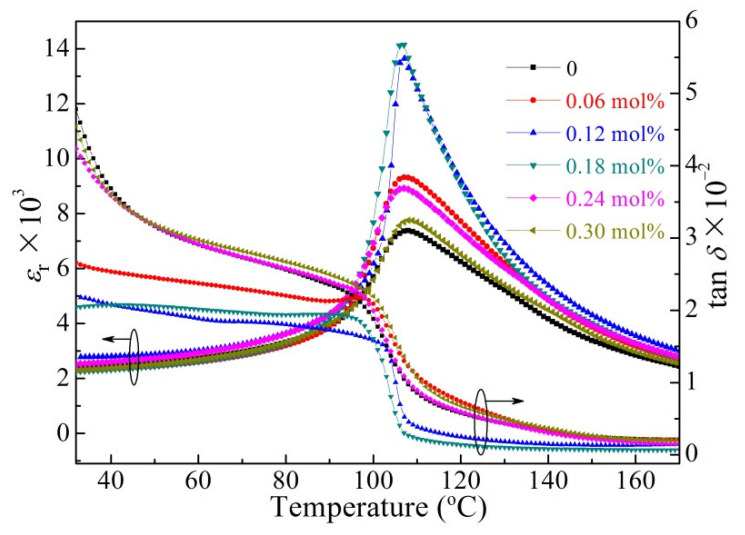
Loss tangents (tan *δ*) (*ε*_r_) and relative permittivities of the BCTZ-NY*x* ceramics with different yttrium contents at 10 kHz.

Temperature-dependences of the relative permittivities (*ε*_r_) and loss tangents (tan *δ*) of the BCTZ-NY*x* ceramics at a frequency of 10 kHz are shown in Figure 3. The cubic-to-tetragonal phase transition temperature (*T*_C_) first decreased and then increased with increasing yttrium content (*x*) (Table S2), whereas the value of *ε*_r_ values presented an opposite trend. The optimum relative permittivity (*ε*_r_ = 14201) was observed for the sample with 0.18 mol% yttrium. Although the grain size might not be the primary reason for the differences in the dielectric properties (Figure 2), the results could be associated with the amount of oxygen vacancies, defect dipoles, and/or structural distortions in the structure, which first decrease at low *x* and then increase at higher *x* [30,31]. The maximum relative dielectric peak of the ceramics first narrowed and then broadened with the increment of yttrium dopant content. The narrowing of the maximum dielectric peak can be associated with limited structural disorder at low *x*, whereas the emergence of large compositional fluctuations and structural defects at excessive doping (high *x*) could broaden the peak [32]. For similar reasons, the loss tangent exhibited an opposite variation tendency to that of the permittivity. The loss tangent of the ceramic was low (<0.025) at a low yttrium content of 0.18 mol%, implying that the sample contained fewer oxygen vacancies and defects. The temperature-dependence of *ε*_r_ at various frequencies of the BCTZ-NY*x* ceramics with different *x* is presented in Figure S3; the dielectric relaxation behaviour first diminished and then enhanced with increment of *x*, suggesting the diffusive phase transition decreased at first and then increased [33,34]. The larger diffusive phase transition at an excessive yttrium content can be attributed to the many valence differences of the elements in the original ABO_3_ structure, leading to the formation of polar nanoregions [35].

**Figure 4:**
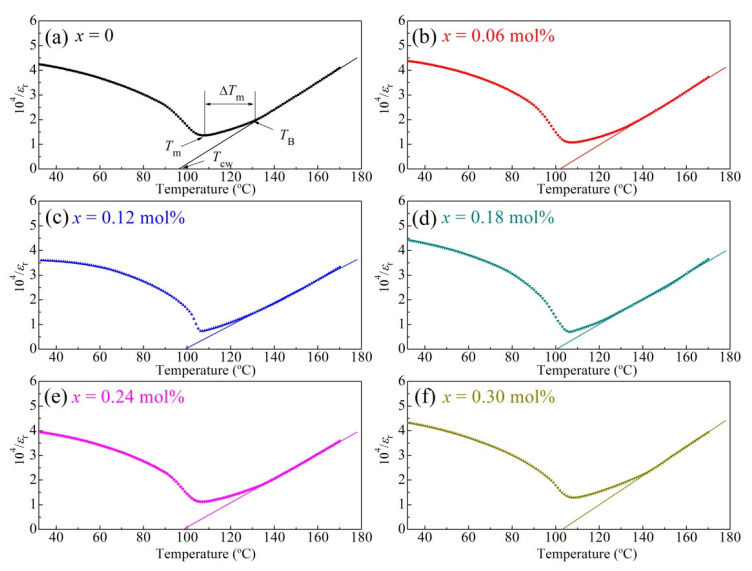
Temperature-dependences of the inverse permittivity (10^4^/*ε*_r_) of the BCTZ-NY*x* ceramics with varying yttrium contents (*x*) of (**a**) 0, (**b**) 0.06, (**c**) 0.12, (**d**) 0.18, (**e**) 0.24, and (**f**) 0.30 mol% at a frequency of 10 kHz.

To characterise the diffuseness of the phase transition, Curie–Weiss law (Equations (1) and (2)) was used to calculate the quantitative parameters of the BCTZ-NY*x* ceramics.

(1) $$\frac{1}{\varepsilon_{r}}=\frac{T-T_{\mathrm{CW}}}{C}\;\;\;(T>\;T_{\mathrm{CW}})$$

(2) $$\Delta T_{m}=T_{B}-T_{m}$$

where *C* is the Curie–Weiss constant, *ε*_r_ represents the relative permittivity, and *T*_CW_ is the Curie–Weiss temperature that is gained by the linear extrapolation of the inverse dielectric constant (10^4^/*ε*_r_) versus temperature curve obtained at 10 kHz (Figure 4). *T*_B_ represents the temperature at which the dielectric constant begins to obey the Curie–Weiss law, *T*_m_ represents the temperature at which the maximum value of the relative dielectric constant emerges, and Δ*T*_m_ represents the degree of deviation from the Curie–Weiss law; it displays the permittivity diffusion degree. All the calculated quantitative parameters are presented in Figure 4 and are also listed in Table S2. Clearly, Δ*T*_m_ first decreased from 23.4 to 18.9 °C and then elevated to 30.4 °C with the increment of yttrium content, indicating that diffusion was limited at low doping contents and then enhanced with excessive doping. The results imply that the yttrium content could influence the diffusive phase transition of the ceramic. This could be associated with the variation in the imbalanced local charges in the structure owing to different defects at different yttrium contents [36]. The diffuseness was enhanced as the long-range ordering dipole was interrupted by excessive yttrium doping, and this could mainly deteriorate the ferroelectricity of the material [37,38]. Similar inferences could be drawn from the fitted diffuseness exponent (Table S2) obtained via a modified Curie–Weiss in Figure S4.

**Figure 5:**
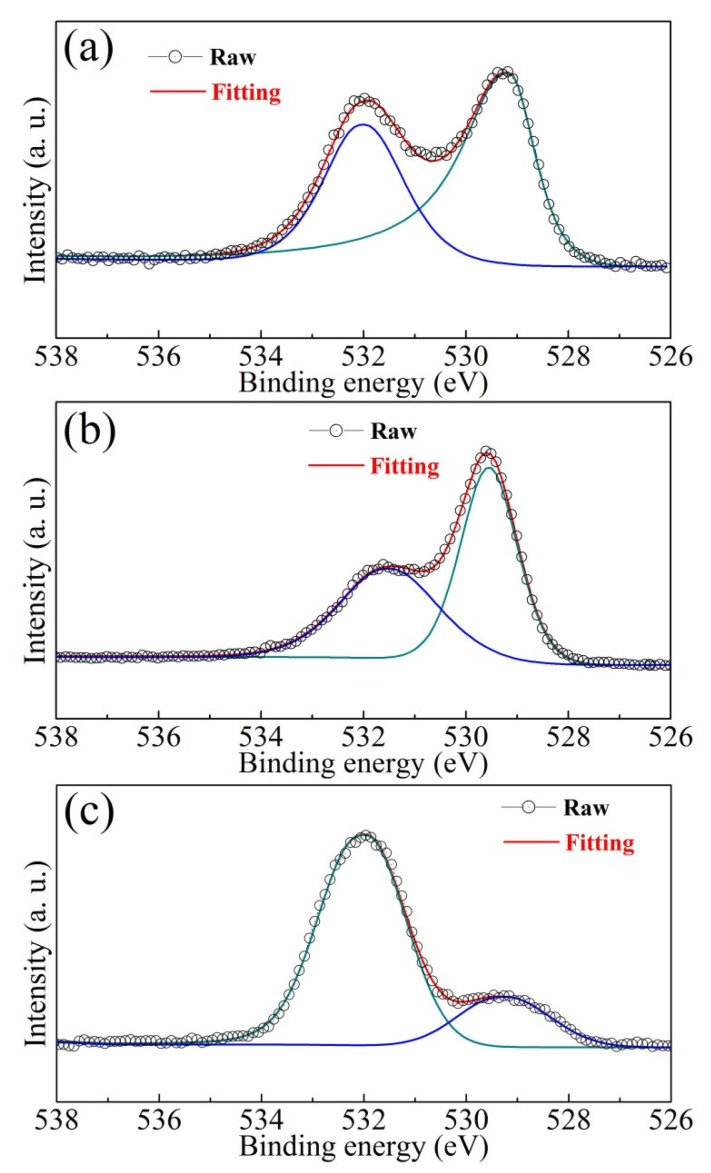
O *1s* valence states in the BCTZ-NY*x* ceramics doping with yttrium contents (*x*) of (**a**) 0, (**b**) 0.18, and (**c**) 0.30 mol%.

In order to demonstrate the formation mechanism of the oxygen vacancies and defect dipoles, XPS analysis was put into effect to determine the different elements electron binding energies in the BCTZ-NY*x* ceramics (Figure 5). Two peaks are observed around the asymmetrical O *1s* photoelectron peak of ~530 eV; the peak at ~531.8 eV represents the oxygen vacancies as adsorbed water at the surface, and the other peak at ~529.4 eV represents cation–oxygen bonds [39,40,41]. The area of the ~531.8 eV peak first decreased with increasing yttrium content from 0 to 0.18 mol% and then increased with a further increment of *x*, indicating that the amount of oxygen vacancies decreased at low *x* and then increased at higher *x* (excessive doping). According to the analysis of the substituted sites in the ABO_3_ structure (Figure 1) and XPS analysis, the possible formation mechanism (defect chemistry) of oxygen vacancies is illustrated in Equations (3) and (4) [42]. The formation mechanism of defect dipoles in samples with abundant yttrium is shown in Equation (5); they strengthen the carrier migration and electron scattering, leading to enhanced dielectric loss [43]. This discussion is in line with the dielectric properties presented in Figure 2.

(3) $$2Y^{3+}+V_{O}^{\cdot \cdot }+\frac{1}{2}O_{2}\left(g\right)\overset{\mathrm{BCZT}-\mathrm{NY}x}{\to }2Y_{\mathrm{Ba}/\mathrm{Ca}}^{\cdot }+O_{O}^{\times }$$

(4) $$2Y^{3+}+O_{O}^{\times }\overset{\mathrm{BCZT}-\mathrm{NY}x}{\to }2Y_{\mathrm{Ti}/\mathrm{Zr}}^{\prime }+V_{O}^{\cdot \cdot }+\frac{1}{2}O_{2}\left(g\right)\;↑$$

(5) $$4Y_{\mathrm{Ti}/\mathrm{Zr}}^{\prime }+2V_{O}^{\cdot \cdot }⇌\left(3Y_{\mathrm{Ti}/\mathrm{Zr}}^{\prime }-V_{O}^{\cdot \cdot }\right)\prime +{\left(Y_{\mathrm{Ti}/\mathrm{Zr}}^{\prime }-V_{O}^{\cdot \cdot }\right)}^{\cdot }$$

### 3.3. Activation Energy

**Figure 6:**
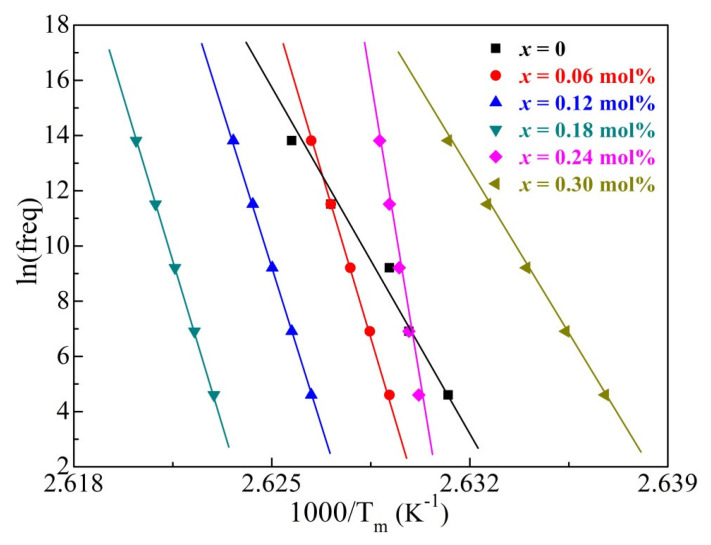
Plots of ln(frequency) versus 1000/*T*_m_ for the BCTZ-NY*x* ceramics.

To further investigate the influence of oxygen vacancies and/or defect dipoles on the domain-switching activation energy of the BCTZ-NY*x* ceramics, the empirical relationship of the Vogel–Fulcher (Equation (6)) was invoked.

(6) $$f=f_{0}\exp\left(\frac{-E_{a}}{k\left(T_{m}-T_{f}\right)}\right)$$

where *E*_a_ represents the activation energy, *T_f_* is the freezing temperature, *k* represents the Boltzmann constant, *f*_0_ represents the pre-exponential factor, and *f* is the measurement frequency. The fitted parameters obtained from the slope of the ln(*f*) versus 1000/*T*_m_ curve are shown in Figure 6 and Table 2. One can observe that, with increasing yttrium contents, *E*_a_ first decreased from 0.676 to 0.401 eV because of the decreased internal stress owing to the reduced structural defects and then increased to 0.633 eV with the re-emergence of oxygen vacancies and defect dipoles (Equations (4) and (5)) [44]. The low diffuseness exponent (1.203) and moderate *E*_a_ (0.401 eV) of the ceramic with 0.18 mol% yttrium suggest that polar nanoregions were rare in the structure. However, the *E*_a_ values were relatively high at excessive *x*, suggesting that polar nanoregions formed owing to structural perturbance induced by the dopant, which is consistent with the analysis in Figure S3 [45]. The fitted value of *T_f_* increased slightly from 106.5 to 107.9 K as the yttrium content increased from 0 to 0.18 mol%, indicating that dipoles could turnover easily and that they were frozen into a glassy state at low *x* [46].

### 3.4. Ferroelectricity

**Figure 7:**
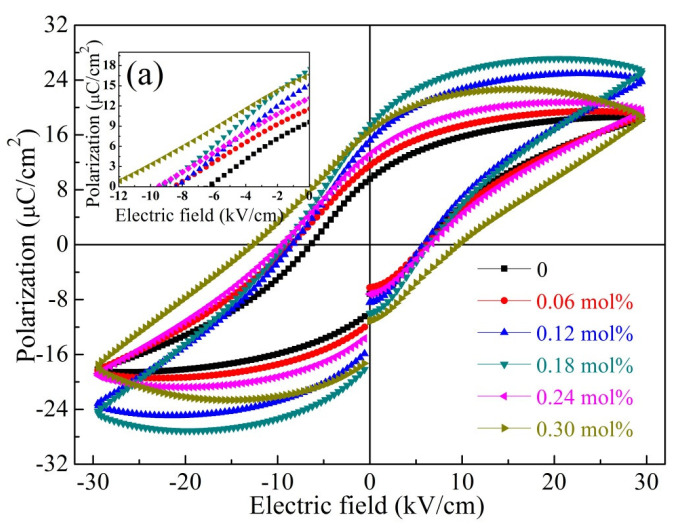
The hysteresis loops of polarisation–electric field (*P-E*) of the BCTZ-NY*x* ceramics; the inset (**a**) displays the *P-E* loops of the selected amplified regions (−12 to 0 kV/cm).

**Figure 8:**
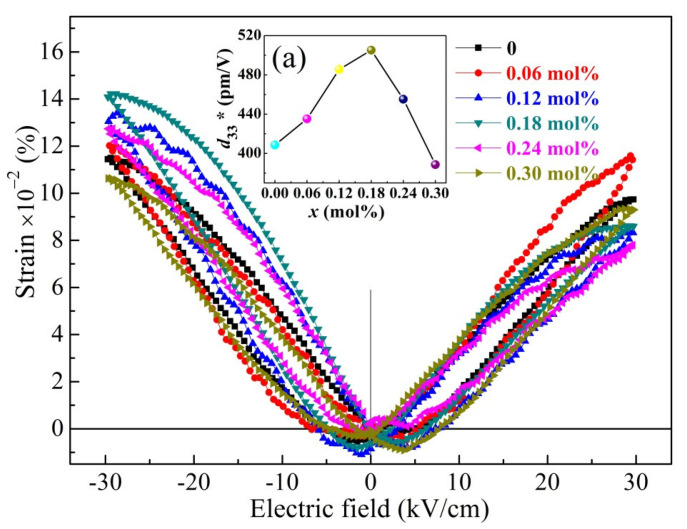
The loops of strain–electric field (*S-E*) of the BCTZ-NY*x* ceramics; the inset (**a**) shows the piezoelectric coefficients (*d*_33_*).

The loops of polarisationelectric field (*P*-*E*) hysteresis of the BCTZ-NY*x* ceramics with various yttrium contents (*x*) are presented in Figure 7. The applied test conditions for all the ceramics were an external electric field of 30 kV/cm and a hysteresis period of 100 ms. The *P*-*E* loops presented a well-saturated hysteresis, and the remnant polarisation (*P*_r_) value first increased from 9.03 (*x* = 0) to 17.65 μC/cm^2^ (*x* = 0.18 mol%) and then decreased with a further increase in *x*, indicating that ferroelectricity was enhanced at low *x* and then deteriorated with a further increase in *x.* Meanwhile, the coercive field (*E*_c_) exhibited an opposite tendency to *P*_r_; this is due to the dipole polarisation rotation variability under the external electric field [47]. That is, a low *E*_c_ most likely appeared at small contents of defect dipoles and oxygen vacancies because the defects could accumulate space charges at grain boundaries and clamp the domain wall switching, consistent with the above analysis [48,49]. Figure 8 shows the butterfly-shaped strain–electric field (*S*-*E*) loops coupled with the *P*-*E* loops, and the calculated piezoelectric coefficients (*d*_33_*) are presented in the inset of Figure 8a. The *d*_33_* value first elevated and then reduced with increasing *x* further, and with 0.18 mol% yttrium for the ceramic, a maximum value of 507 pm/V was observed.

### 3.5. Piezoelectricity

**Figure 9:**
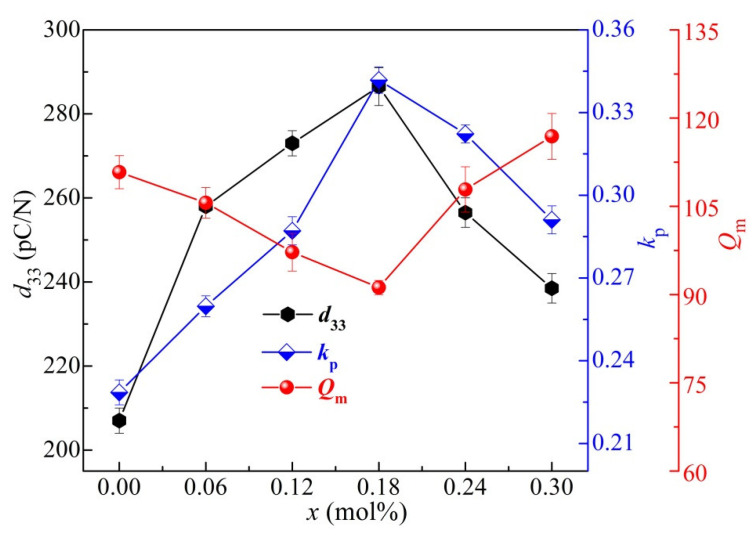
Mechanical quality factor (*Q*_m_), planar vibration electromechanical coupling factors (*k*_p_), and piezoelectric constant (*d*_33_) of the BCTZ-NY*x* ceramics.

The piezoelectric properties (viz., planar vibration electromechanical coupling factors (*k*_p_), mechanical quality factor (*Q*_m_), and piezoelectric constant (*d*_33_)) of the BCTZ-NY*x* ceramics with different yttrium contents (*x*) were evaluated after a DC poling process (30 kV/cm for 40 min at room temperature in a silicone oil bath) followed by a surface charge-elimination process (48 h in air) [50]. All the related parameters are shown in Figure 9. Evidently, *d*_33_ and *k*_p_ first increased at low *x* and then reduced with a further increasing *x*, while *Q*_m_ presented an opposite tendency. The elevated piezoelectric properties can be due to two factors: first, the doping of Y^3+^ as a donor in the ABO_3_ structure (A sites; valence: +2) could decrease the number of defect dipoles and oxygen vacancies (Equation (3)) and thus reduce the internal stress [51], which was also confirmed by the coefficients of thermal expansion (*CTE*) in Figure S5. Second, many covalent bonds with *sp*^3^ hybridisation were formed because the highly electronegative Y^3+^ (1.22) ion substituted the B site Ba^2+^ (1.00) and/or Ca^2+^ (0.89) ions in the ABO_3_ structure [52]. At *x* = 0.18 mol% of the ceramic, the optimal values of *k*_p_, *d*_33_, and *Q*_m_ were 0.34, 284 pC/N, and 91, respectively. Apparently, the BCTZ-NY*x* ceramics in this study featured relatively outstanding piezoelectric and ferroelectric properties, compared with the state-of-the-art BCZT, Bi_0.5_Na_0.5_TiO_3_, BiFeO_3_, and K_0.5_Na_0.5_NbO_3_ lead-free piezoelectric ceramics [7,8,21,53].

## 4. Conclusions

Environmentally friendly lead-free ceramics of Ba_0.90_Ca_0.10_Ti_0.96_Zr_0.04_O_3_-0.05 mol%Nd-*x*Y (*x* = 0–0.30 mol%) were synthesised by a solid-state reaction from as-prepared nanoparticles. The phase structure feature, electrical properties, and fracture morphology were researched to evaluate their piezoelectric performance. The ceramics featured high relative densification (~96%) and high fracture strengths (~90 MPa), indicating that the preparation process was effective. At low doping levels, yttrium could take up A sites in the ABO_3_ structure, leading to increased crystalline interplanar spacing, donor doping characteristics, and reduced oxygen vacancies, enhancing dielectric, piezoelectric, and ferroelectric properties. At *x* = 0.18 mol%, the lowest domain-switching activation energy (0.401 eV) was found because of the decrease in the internal stress owing to the reduced number of defect dipoles in the structure. However, at high *x*, the electrical properties deteriorated owing to structural defects caused by excessive doping. The excellent thermophysical performances and piezoelectric properties (*d*_33_ = 284 pC/N and *d*_33_* = 507 pm/V) of the ceramics suggest that they are good candidates for the materials of sensors and transducers in electronic components.

## Figures and Tables

**Table 1:** Lattice parameters (*a*, *b*, *c*, and *Axial angle*) and densification (density (*ρ*) and relative density (*ρ*_r_)) of the BCTZ-NY*x* ceramics with various yttrium contents (*x*).

*x* (mol%)	*a* (Å)	*b* (Å)	*c* (Å)	*Axial Angle* (^o^)	*ρ* (g/cm^−3^)	*ρ*_r_ (%)
0	4.013(3)	4.013(3)	4.013(3)	89.05	5.491	95.54
0.06	4.015(1)	4.015(1)	4.015(1)	88.96	5.517	96.00
0.12	4.018(2)	4.018(2)	4.018(2)	88.89	5.536	96.34
0.18	4.022(0)	4.022(0)	4.022(0)	88.85	5.541	96.42
0.24	4.017(8)	4.017(8)	4.017(8)	88.97	5.533	96.27
0.30	4.014(7)	4.014(7)	4.014(7)	89.01	5.529	96.10

**Table 2:** Activation energy (*E*_a_), freezing temperature (*T*_f_), and pre-exponential factor (*f*_0_) of the BCTZ-NY*x* ceramics.

*x* (mol%)	*E*_a_ (eV)	*T_f_* (K)	*f*_0_ (×10^21^ Hz)
0	0.676	106.5	5.32
0.06	0.584	106.9	6.75
0.12	0.475	107.3	7.63
0.18	0.401	107.9	8.44
0.24	0.527	106.9	7.69
0.30	0.633	105.8	5.84

## Data Availability

Data is available from the corresponding author upon reasonable request.

## Supplementary Materials

The following supporting information can be downloaded at: https://www.mdpi.com/article/10.3390/ma16062369/s1, Figure S1: Rietveld refinement of the XRD pattern of the BCTZ-NY*x* ceramic with 0.18 mol% yttrium belonging to the *R*3m space group (rhombohedral structure) using Fullprof software. The cross indicates the experimental intensity, the red line represents the calculated pattern, the blue vertical line shows the Bragg position, and the magenta line represents the difference plot; Figure S2: Raman spectra of the BCTZ-NY*x* ceramics with different yttrium contents (*x*); Figure S3: Temperature-dependence of the relative permittivity (*ε*_r_) and loss tangent (tan *δ*) of the BCTZ-NY*x* ceramics with yttrium contents (*x*) of (**a**) 0, (**b**) 0.06, (**c**) 0.12, (**d**) 0.18, (**e**) 0.24, and (**f**) 0.30 mol% under different measuring frequencies; Figure S4: Plots of ln(1/*ε*_r_ − 1/*ε*_m_) versus ln(*T* − *T*_m_) of the BCTZ-NY*x* ceramic at a frequency of 10 kHz; Figure S5: Coefficient of thermal expansion (CTE) of the BCTZ-NY*x* ceramic with an yttrium content of 0.18 mol% with increasing temperature; Table S1: Coefficient of thermal expansion (*CTE*; *CTE*_1_ line at temperatures below 70 °C and *CTE*_2_ line at temperatures above 200 °C) and fracture strength (*K*) of the BCTZ-NY*x* ceramics; Table S2: Curie–Weiss temperature (*T*_CW_), temperature at which the permittivity begins to follow the Curie–Weiss law (*T*_B_), temperature deviation (Δ*T*_m_), Curie–Weiss constant (*C*), and diffuseness exponent (*γ*) of the BCTZ-NY*x* ceramic as a function of the yttrium content (*x*) at 10 kHz. References [29,54] are cited in the supplementary materials.
