# Supplementary material for: Piezoelectricity and Thermophysical Properties of Ba0.90Ca0.10Ti0.96Zr0.04O3 Ceramics Modified with Amphoteric Nd3+ and Y3+ Dopants
Source: Materials (Basel). 2023 Mar 15;16(6):2369. doi: 10.3390/ma16062369 (PMC10052513; doi:10.3390/ma16062369)
Supplement: Supplementary file 1 [file materials-16-02369-s001.zip › materials-2233252-supplementary.pdf]

Supplementary Information

Piezoelectricity and Thermophysical Properties of $Ba_{0.90}Ca_{0.10}Ti_{0.96}Zr_{0.04}O_3$ Ceramics Modified with Amphoteric Nd^{3+} and Y^{3+} Dopants

Yongshang Tian [1,*], Mingyang Ma [1], Shuiyun Li [1], Junli Dong [1], Xiang Ji [2], Haitao Wu [3], Jinshuang Wang [1] and Qiangshan Jing [1,*]

[1] College of Chemistry and Chemical Engineering, Henan Key Laboratory of Utilization of Non-Metallic Mineral in the South of Henan, Xinyang Normal University, Xinyang 464000, China

[2] School of Materials and Chemical Technology, Tokyo Institute of Technology, Tokyo 152-8552, Japan

[3] School of Environmental and Material Engineering, Yantai University, Yantai 264005, China

* Correspondence: tianyongshang@xynu.edu.cn or tianyongshang423@163.com (Y.T.); 9jqshan@163.com (Q.J.); Tel.: +86–0376–6390603 (Y.T.)

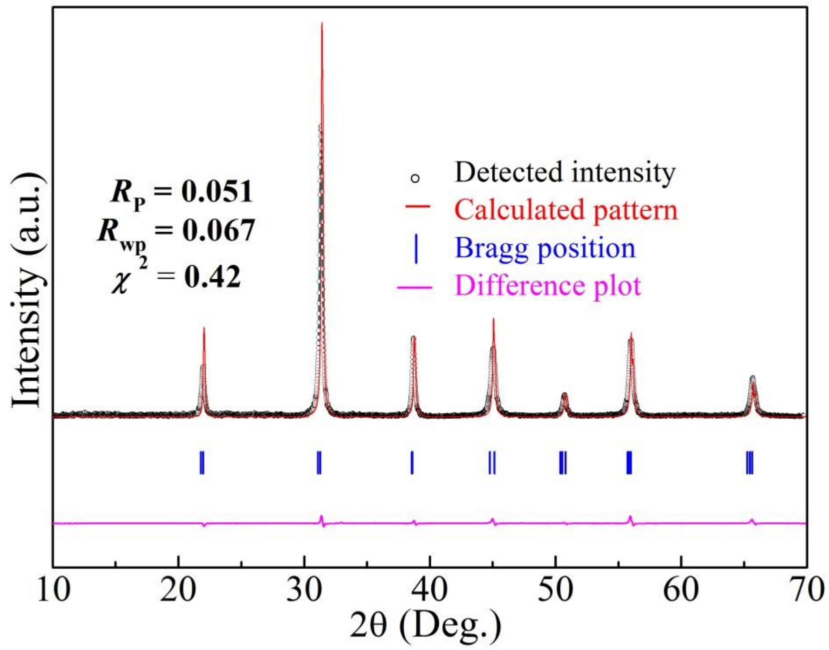

Figure S1. Rietveld refinement of the XRD pattern of the BCTZ-NY*x* ceramic with 0.18 mol% yttrium belonging to the *R*3m space group (rhombohedral structure) using Fullprof software. The cross indicates the experimental intensity, the red line represents the calculated pattern, the blue vertical line shows the Bragg position, and the magenta line represents the difference plot.

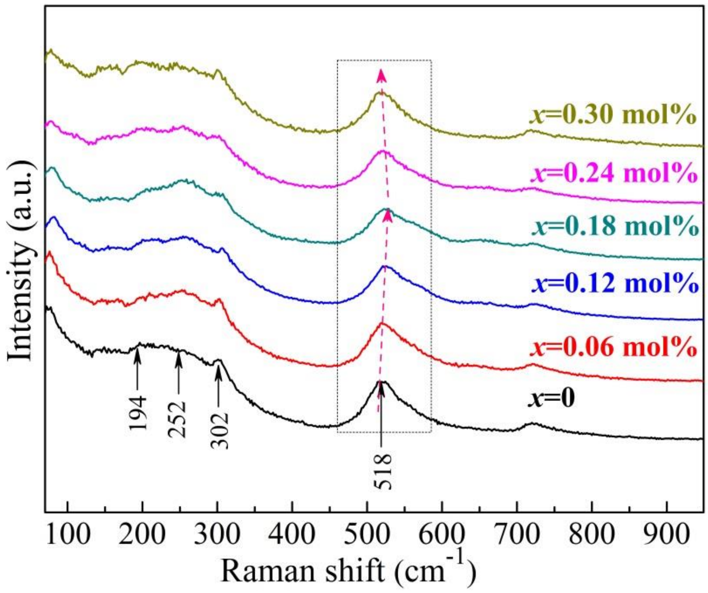

Figure S2. Raman spectra of the BCTZ-NY*x* ceramics with different yttrium contents (*x*).

Table S1. Coefficient of thermal expansion (*CTE*; CTE_1 line at temperatures below 70 °C and CTE_2 line at temperatures above 200 °C) and fracture strength (*K*) of the BCTZ-NY*x* ceramics.

x (mol%)	CTE_1 ($\times10^{-5}$ K^{-1})	CTE_2 ($\times10^{-5}$ K^{-1})	*K* (MPa)
0	0.98	1.35	84.9
0.06	1.01	1.39	88.5
0.12	1.02	1.42	92.6
0.18	1.03	1.46	96.3
0.24	1.03	1.44	94.0
0.30	1.01	1.40	89.7

Table S1 shows the fracture strength (*K*) values of the BCTZ-NY*x* ceramics with different yttrium contents. Although the thermal expansion characteristic could impact the service life of the BCTZ–NYx ceramic, the fracture strength (*K*) of a ceramic could affect its long-term usage by preventing the formation of cracks. *K* first increased and then decreased monotonously with increasing Y^{3+} content; this is associated with the initial decrease in the number of structural defects followed by an increase with increasing yttrium content, as discussed in the main manuscript. Moreover, the ceramics prepared in this study exhibit relatively higher fracture strengths than those reported earlier [S1,S2], which can be attributed to their high densification and low structural defects (Figure 2).

The characteristic Raman vibrations at ~302, 518, and 719 cm^{-1} indicate that the BCTZ-NY*x* ceramic had a pure perovskite structure (ABO_3) with a rhombohedral structure. The characteristic vibrational peaks initially shifted to higher wavenumbers and then to lower wavenumbers because the distance between the atoms in the oxygen octahedra first decreased and then increased with increasing yttrium substitution in the structure, which is consistent with the cell parameters determined by the Rietveld fitting procedure presented in Table 1. Moreover, the slight broadening of the Raman peak at ~518 cm^{-1} implies that the short-range polarisation mismatch was enhanced with excessive yttrium doping.

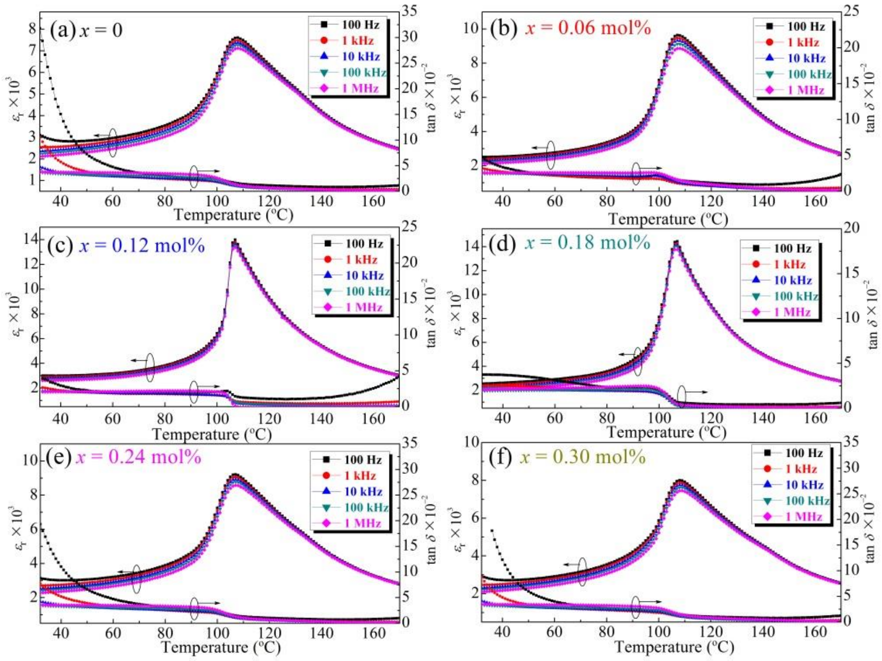

Figure S3. Temperature-dependence of the relative permittivity (ε_r) and loss tangent (tan δ) of the BCTZ-NY*x* ceramics with yttrium contents (*x*) of (**a**) 0, (**b**) 0.06, (**c**) 0.12, (**d**) 0.18, (**e**) 0.24, and (**f**) 0.30 mol% under different measuring frequencies.

Table S2. Curie–Weiss temperature (T_{CW}), temperature at which the permittivity begins to follow the Curie–Weiss law (T_B), temperature deviation (ΔT_m), Curie–Weiss constant (C), and diffuseness exponent (γ) of the BCTZ-NYx ceramic as a function of the yttrium content (x) at 10 kHz.

x (mol%)	T_{CW} (°C)	T_m (°C)	T_B (°C)	ΔT_m (°C)	C (×10^5)	γ
0	96.2	108.1	131.5	23.4	1.816	1.470
0.06	101.9	107.2	129.7	22.5	1.895	1.595
0.12	99.0	107.0	127.2	20.2	2.173	1.226
0.18	100.3	106.9	125.8	18.9	2.205	1.203
0.24	98.1	107.4	132.3	24.9	2.024	1.461
0.30	102.9	108.3	138.7	30.4	1.701	1.488

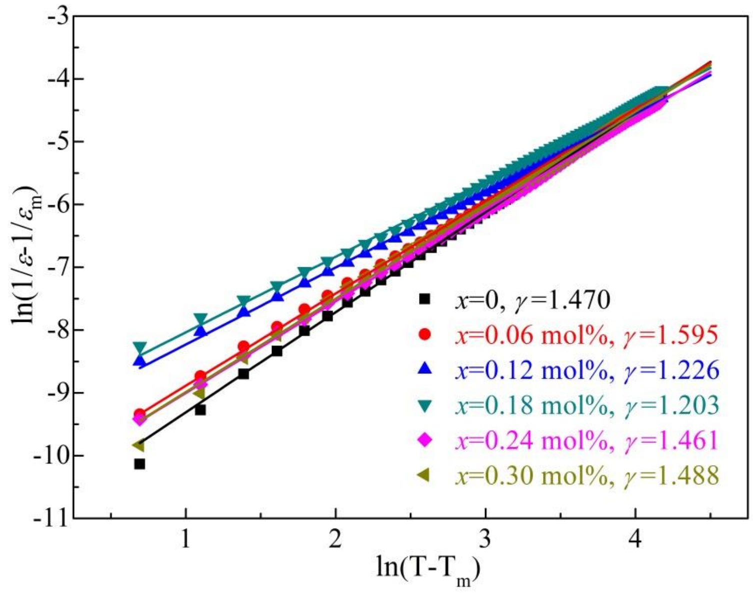

Figure S4. Plots of $\ln(1/\varepsilon_r - 1/\varepsilon_m)$ versus $\ln(T - T_m)$ of the BCTZ-NYx ceramic at a frequency of 10 kHz.

The modified Curie–Weiss law (Equation S1) was used to describe the dielectric relaxation behaviour of the BCTZ-NYx ceramics.

$$\frac{1}{\varepsilon_r} - \frac{1}{\varepsilon_m} = \frac{(T - T_m)^\gamma}{C'} \tag{S1}$$

where ε_m is the maximum relative permittivity, C' is the modified Curie–Weiss constant, and γ is the diffuseness exponent; γ ranges from 1 for a normal ferroelectric to 2 for a typical relaxor ferroelectric. γ was calculated from the slope of the $\ln(1/\varepsilon_r - 1/\varepsilon_m)$ versus $\ln(T - T_m)$ curve at 10 kHz, and the results are shown in Figure S4 and Table S2. The γ value decreased from 1.470 to 1.203 with a small increase in the yttrium content, suggesting that the ferroelectricity of the ceramic was improved and the diffusive phase transition was impeded. However, the γ value increased to 1.488 with a further increase in the yttrium content, indicating that the diffusive phase transition was enhanced.

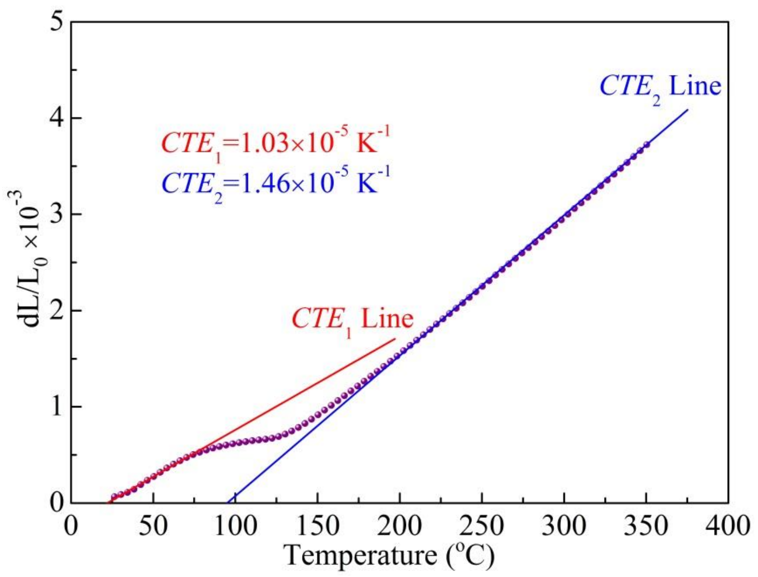

Figure S5. Coefficient of thermal expansion (CTE) of the BCTZ-NY*x* ceramic with an yttrium content of 0.18 mol% with increasing temperature.

The coefficient of thermal expansion (*CTE*) of the BCTZ-NY*x* ceramic with an yttrium content of 0.18 mol% was determined via fitting analyses, as shown in Figure S5. The linear CTE_1 plot at temperatures below 70 °C and the linear CTE_2 plot at temperatures above 200 °C suggest that the lattice expansion behaviour of the ceramic varied with the temperature. The CTE_2 values were larger than the CTE_1 ones because the thermal expansion of the paraelectric phase of the ceramic was higher than that of the ferroelectric phase. The fitted *CTE* values for the low (<70 °C) and high temperature (>200 °C) ranges are shown in Table S1. The thermal expansion first increased and then decreased with increasing yttrium content. This is because of the decrease in structural defects and internal stress at low yttrium contents and the re-emergence of the structural defects at high yttrium contents.

References

S1. Xu, Q.; Huang, D.P.; Chen, W.; Zhang, F.; Wang, B.T. Structure, electrical conducting and thermal expansion properties of $Ln_{0.6}Sr_{0.4}Co_{0.2}Fe_{0.8}O_3$ (Ln = La, Pr, Nd, Sm) perovskite-type complex oxides. *J. Alloy. Comp.* **2007**, *429*, 34–39. https://doi.org/10.1016/j.jallcom.2006.04.005.

S2. Adhikari, P.; Mazumder, R.; Abhinay, S. Electrical and mechanical properties of MgO added $0.5Ba(Zr_{0.2}Ti_{0.8})O_3$–$0.5(Ba_{0.7}Ca_{0.3})TiO_3$ (BZT–0.5BCT) composite ceramics. *J. Electroceram.* **2016**, *37*, 127–136. https://doi.org/10.1007/s10832-016-0049-7.
